# Lactic Acid Preparations

**Published:** 1909-06-19

**Authors:** 


### LACTIC ACID PREPARATIONS.

There has lately been so strong a development of the lactic acid bacillary treatment of gastro-intestinal affections that some definite criteria would seem to be essential by which to judge whether a given proprietary lactic acid preparation is likely to be of any value or not. We need not enter into a detailed discussion of the *raison d'être* of the treatment itself; The remarks made by Dr. Herschell in his book upon the subject, recently reviewed by us, summarise present-day views upon the matter concisely.

The first difficulty confronting the medical man who wishes to make use of lactic acid bacilli or their products as therapeutic agents is the selection of the best preparation from amongst the number now upon the market, some of which are good, some useless, some positively harmful. There are at least five different types, each represented by various species. They are (1) Yoghourt prepared with the Maya ferment; (2) milks that have been soured ready for use; (3) liquid cultures of bacilli for internal administration supplied in bottles; (4) liquid cultures to be used for souring the milk at home, usually supplied in phials or in tubes; and (5) dried cultures supplied in the forms of either powders or tablets, to be used either for souring milk that is to be consumed, or else for internal administration just as supplied.

It seems to be generally allowed—and Dr. Herschell has tested the point and proved it—that the dry preparations are better than those that are liquid. At the same time many even of the dry preparations are relatively inefficient, especially when the tablet is not readily soluble in water. Preparations that effervesce are to be received with considerable caution, because one of the devices for masking the insolubility of the base of which a tablet is composed is to mix an effervescing powder with it. This certainly causes the mass to disintegrate, but the solubility is apt to be more apparent that real, to say nothing of the fact that the salt and the acid used to produce effervescence are more than likely to interfere with the proper growth of any lactic acid bacilli that may be present.

As a practical guide in the selection of the tablet or the powder to employ, Dr. Herschell lays down the following rules:

*In the case of a Tablet.*—(1) It should be pure white in colour. (2) It should be odourless, both when in the dry state and when moistened with water. (3) When immersed it should disintegrate into a fine powder within three minutes without the aid of crushing or stirring, and this disintegration should not be accompanied by effervescence. (4) The suspension should be neutral to litmus paper. (5) When cultivated on Cohendy's serum or other suitable medium it should give an abundant growth of the Bulgarian bacillus (the bacillus of Boucard), and no other micro-organism should be present.

*In the case of a powder for the souring of milk.*—(1) It should produce a good firm clot upon pure milk in eight hours at a temperature of 100° F. (2) The clot should be nearly white, spongy, with a faintly acid smell and taste, but without any acridity. (3) No brown sediment should form during the incubation. (4) The only organisms present in the soured milk should be the Bulgarian bacillus, together with one other organism that is added to prevent the saponification of the milk fat.

It need scarcely be added that many advertised preparations fail to comply with these simple tests.

